# Engineering *Saccharomyces cerevisiae* for production of the capsaicinoid nonivamide

**DOI:** 10.1186/s12934-022-01831-3

**Published:** 2022-05-28

**Authors:** Nina Muratovska, Carl Grey, Magnus Carlquist

**Affiliations:** 1 Division of Applied Microbiology, Department of Chemistry, Faculty of Engineering, Lund University, Lund, Sweden; 2 Division of Biotechnology, Department of Chemistry, Faculty of Engineering, Lund University, Lund, Sweden

**Keywords:** Capsaicinoids, Capsaicin, Chilli pepper, *Capsicum*, Fatty acids, Nonanoic acid, TRPV1 agonist, Yeast, Vanillylamine, *N*-Acyltransferase, CoA-ligase, Whole-cell bioconversion

## Abstract

**Background:**

Capsaicinoids are produced by plants in the *Capsicum* genus and are the main reason for the pungency of chili pepper fruits. They are strong agonists of TRPV1 (the transient receptor potential cation channel subfamily V member 1) and used as active ingredients in pharmaceuticals for the treatment of pain. The use of bioengineered microorganisms in a fermentation process may be an efficient route for their preparation, as well as for the discovery of (bio-)synthetic capsaicinoids with improved or novel bioactivities.

**Results:**

*Saccharomyces cerevisiae* was engineered to over-express a selection of amide-forming *N*-acyltransferase and CoA-ligase enzyme cascades using a combinatorial gene assembly method, and was screened for nonivamide production from supplemented vanillylamine and nonanoic acid. Data from this work demonstrate that Tyramine *N-*hydroxycinnamoyl transferase from *Capsicum annuum* (CaAT) was most efficient for nonivamide formation in yeast, outcompeting the other candidates including AT3 (Pun1) from *Capsicum* spp. The CoA-ligase partner with highest activity from the ones evaluated here were from *Petunia hybrida* (PhCL) and *Spingomonas* sp.* Ibu-2* (IpfF). A yeast strain expressing CaAT and IpfF produced 10.6 mg L^−1^ nonivamide in a controlled bioreactor setup, demonstrating nonivamide biosynthesis by *S. cerevisiae* for the first time.

**Conclusions:**

Baker’s yeast was engineered for production of nonivamide as a model capsaicinoid, by expressing *N*-acyltransferases and CoA-ligases of plant and bacterial origin. The constructed yeast platform holds potential for in vivo biocatalytic formation of capsaicinoids and could be a useful tool for the discovery of novel drugs.

**Supplementary Information:**

The online version contains supplementary material available at 10.1186/s12934-022-01831-3.

## Background

**Fig. 1:**
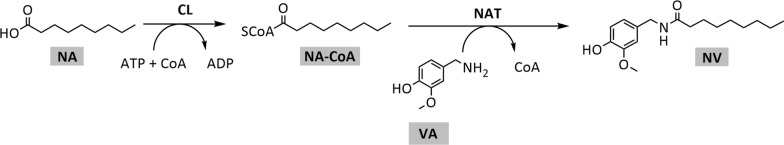
Reactions catalyzed by the enzyme cascade of interest in this work. *NA* nonanoic acid, *NA-CoA *nonanoyl-CoA, *VA* vanillylamine, *NV* nonivamide, *CL* CoA-ligase, *NAT* *N*-acyltransferase

Capsaicinoids are a group of alkaloid compounds found in plants of the *Capsicum* genus. Their structure consists of a vanilloid moiety, and an acyl chain connected by an amide bond (Fig. 1). Several capsaicinoids have been identified in chilli plants, with capsaicin and dihydrocapsaicin being the most abundant [1]. The quantity and composition in different cultivars is affected by variation in cultivation conditions, temperature, and light exposure [2]; therefore production of specific capsaicinoids by plant extraction is challenging and requires extensive purification procedures. These compounds are commonly used for flavouring in the food industry [3], but also have non-food applications such as being active ingredients in pepper sprays [4], bio-repellents [5], and analgesics [6]. The physiological effect of capsaicinoids stems from their strong agonistic effect on the transient receptor potential cation channel subfamily V member 1 (TRPV1) [7]. The TRPV1 receptor plays a major role in nociception in higher eukaryotes and is an important drug target for treatment of pain [8]; therefore novel capsaicinoids with improved drug properties are highly desired.

The use of recombinant baker’s yeast, *Saccharomyces cerevisiae*, engineered for biosynthesis of specific capsaicinoids could be a consistent and sustainable production route for various capsaicinoids; however, this has not been achieved previously. In fact, the complete biosynthesis pathway has not been previously transferred to any recombinant production host. Baker’s yeast is an attractive production host due to its ability to functionally express plant genes, its genetic accessibility using a range of genetic engineering tools, as well as its high robustness to process conditions [9]. Moreover, metabolic engineering of a de novo pathway in yeast may be used as a tool for development of novel capsaicinoids with more beneficial drug properties, e.g. higher specificity towards TRPV1 and less adverse side-effects.

*Saccharomyces cerevisiae* has been successfully engineered for de novo synthesis of the capsaicinoid precursor vanillin [10], and several free fatty acids of various lengths (C6–C18) [11, 12]. In vivo transamination with recombinant amine transaminases (ATA) has also been demonstrated [13, 14], although not yet for production of vanillylamine. A key challenge to reach complete synthesis of capsaicinoids in yeast is to achieve a functional amide-forming step. In chili pepper, capsaicin is believed to be formed by capsaicin synthase (CS), encoded by *AT3* (also named *Pun1*) [15, 16], a coenzyme A-thioester-dependent *N*-acyl transferase (NAT) [17] belonging to the BAHD superfamily [18]. The activated carboxylic donor acyl-CoA substrate can be formed from the corresponding fatty acid by acyl-CoA synthetase (ACS), a coenzyme A ligase (CL) [EC 6.3.1.-] [19, 20]. CLs operate by a rather complex mechanism involving two half reactions separated by a significant conformational change, requiring both CoA-SH and ATP [21]. Transfer of a NAT-CL cascade system to a biocatalytic setting is not straightforward, and requires fine-tuning of conditions to suit both NAT and CL, as well as a system for regeneration of ATP and CoA [22, 23]. The use of NATs for biocatalytic amidation has gained increased attention in recent years [24, 25]. An increasing number of NATs have been characterized, thereby shedding light into their vast diversity in product range, e.g. α,β-unsaturated acid phenethyl amides [26], aliphatic amides [27], acylated anthocyanins [28], and monoterpenoid indole alkaloids [17]. Previously, ten acyltransferases belonging to the BAHD family were expressed in *S. cerevisiae* and used for the synthesis of various hydroxycinnamate and benzoate ester and amide conjugates [29]. A more recent study demonstrated that expressing a BAHD acyltransferase named HDT1, isolated from *Trifolium pratense* (red clover), together with 4-coumarate:CoA ligase (4CL) from *Arabidopsis thaliana*, led to biosynthesis of clovamide and other analogues in *S. cerevisiae* and *Lactococcus lactis* [30].

In this work, a number of NAT and CL enzymes of plant and bacterial origin with previously reported activity for formation of aliphatic amides were evaluated for the production of the model capsaicinoid nonivamide in *S. cerevisiae* (Fig. 1). Furthermore, the potential of yeast was assessed both in terms of production and for its tolerance to capsaicinoids and their precursors.

## Results

### Cell tolerance to capsaicinoids and their precursors

**Fig. 2:**
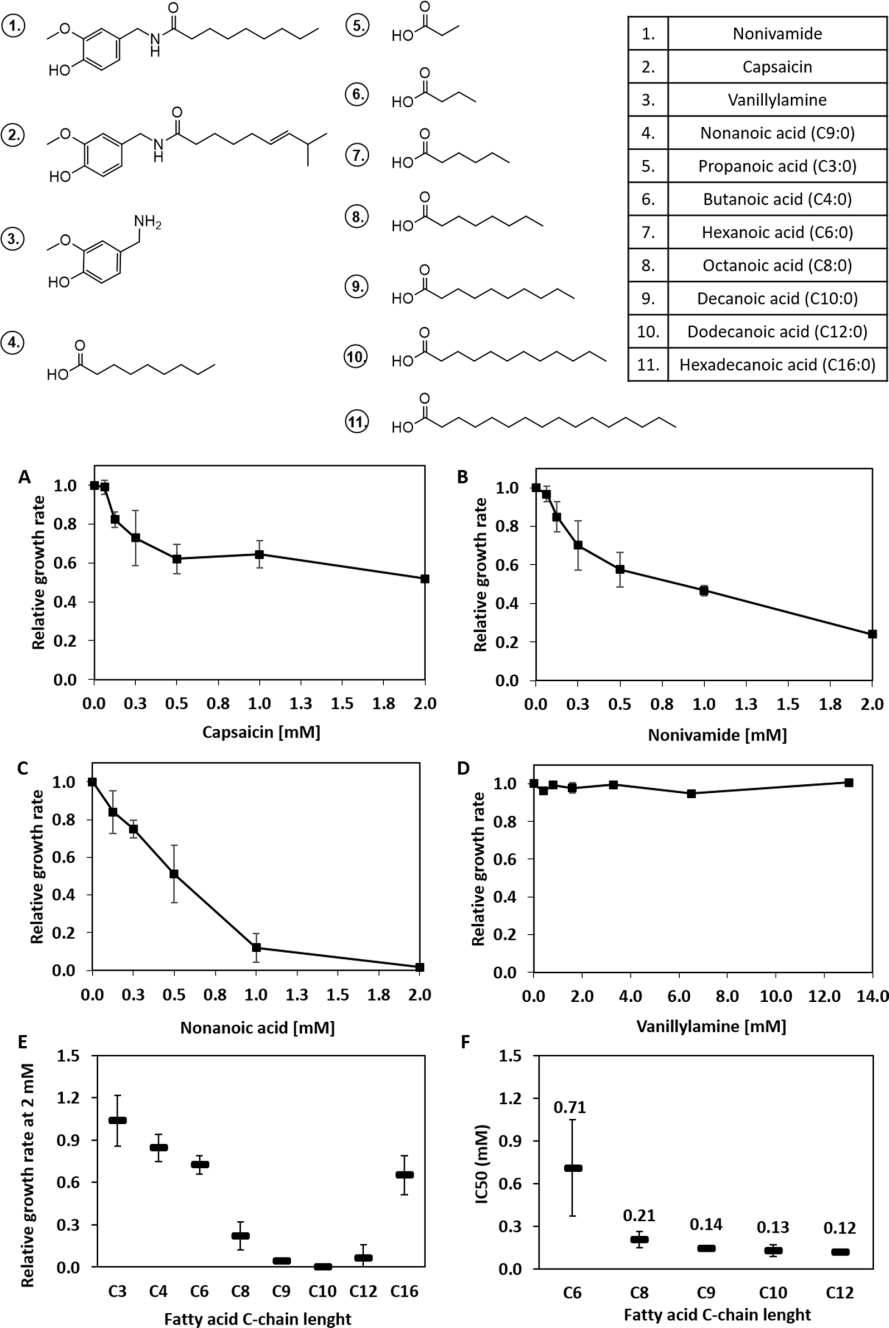
Structures of the various compounds mentioned in this work [1–11]. Relative growth rates when compounds were supplied in the medium in concentrations between 0–2 mM for **A** capsaicin, **B** nonivamide and **C** nonanoic acid and 0–13 mM for **D** vanillylamine. **E** Relative growth rate for the saturated fatty acids at 2 mM. **F** Half inhibitory (IC50) values for the medium chain saturated fatty acids

To shed light on the potential of *S. cerevisiae* as a prospective platform host for production of capsaicinoids, a series of growth inhibition experiments were performed. Nonivamide and capsaicin both gave a significant growth inhibition already at a concentration of 0.5 mM; however, cell growth was not completely arrested within the concentration range investigated (0–2 mM) (Fig. 2A, B). The observed inhibitory concentrations are similar to what was previously found for yeast (0.82–1.64 mM) [31], which in turn is significantly higher than reported for several bacterial species (0.03–0.21 mM) [32, 33]. Vanillylamine was not inhibitory for yeast within the investigated concentrations (up to 13 mM) (Fig. 2D). The manifold higher inhibitory activity of capsaicinoids can therefore be attributed to the aliphatic side-chain functionality or the amphiphilic property, which previously was found to direct the compound into the phospholipid bilayer [34], possibly affecting membrane properties and function. Nonanoic acid was found to be inhibitory already at 0.25 mM and completely hindered growth at 2 mM, thus posing a possible challenge for yeast-based production (Fig. 2C).

To get a more complete picture of the tolerance of yeast to specific fatty acids, and its potential to produce capsaicinoids with different aliphatic chain lengths, the inhibitory activity of an additional number of saturated fatty acids (SFAs) between C2–C16 was evaluated (Fig. 2E). Highest inhibition was observed for the medium-chain FAs octanoic (C8:0), nonanoic (C9:0), decanoic (C10:0) and dodecanoic (C12:0) acid (Fig. 2E). Half maximal inhibitory values (IC50s) for the medium-chain FAs (Fig. 2F) were in agreement with previous studies where hexanoic, octanoic and decanoic acid were found to be inhibitory at a concentration of 1 mM [35]. Acids with shorter-[propanoic (C3:0) and butanoic (C4:0) acid] and longer-[hexadecanoic acid (C16:0)] chain length were less inhibitory.

### Over-expression of *AT3* and *ACS* from *Capsicum* and whole-cell biosynthesis of nonivamide

The capsaicinoid biosynthesis pathway in plantae has been extensively studied previously [36, 37]. The enzymes referred to as being responsible for the amide-forming step are the acyltransferase AT3 and the CoA-ligase ACS [15, 20]. To map their activity in yeast, codon-optimised genes *AT3* and *ACS* from *Capsicum* sp. were synthesised and over-expressed. The prototrophic strain CEN.PK 113-7D was engineered with the CRISPR-Cas9 system to carry the integrated *AT3* and *ACS* under the control of strong constitutive promoters (TEF1p and GPDp) and terminators (PGK1t and ADH1t), generating strain TMBNM020 (Table 1). These were chosen due to their standard use in yeast expression systems and to reach high protein levels throughout the bioconversion. Another strain, TMBNM009, carrying a 2µ multi-copy plasmid with the same *AT3*-*ACS* expression cassette was also constructed (Table 1).

**Fig. 3:**
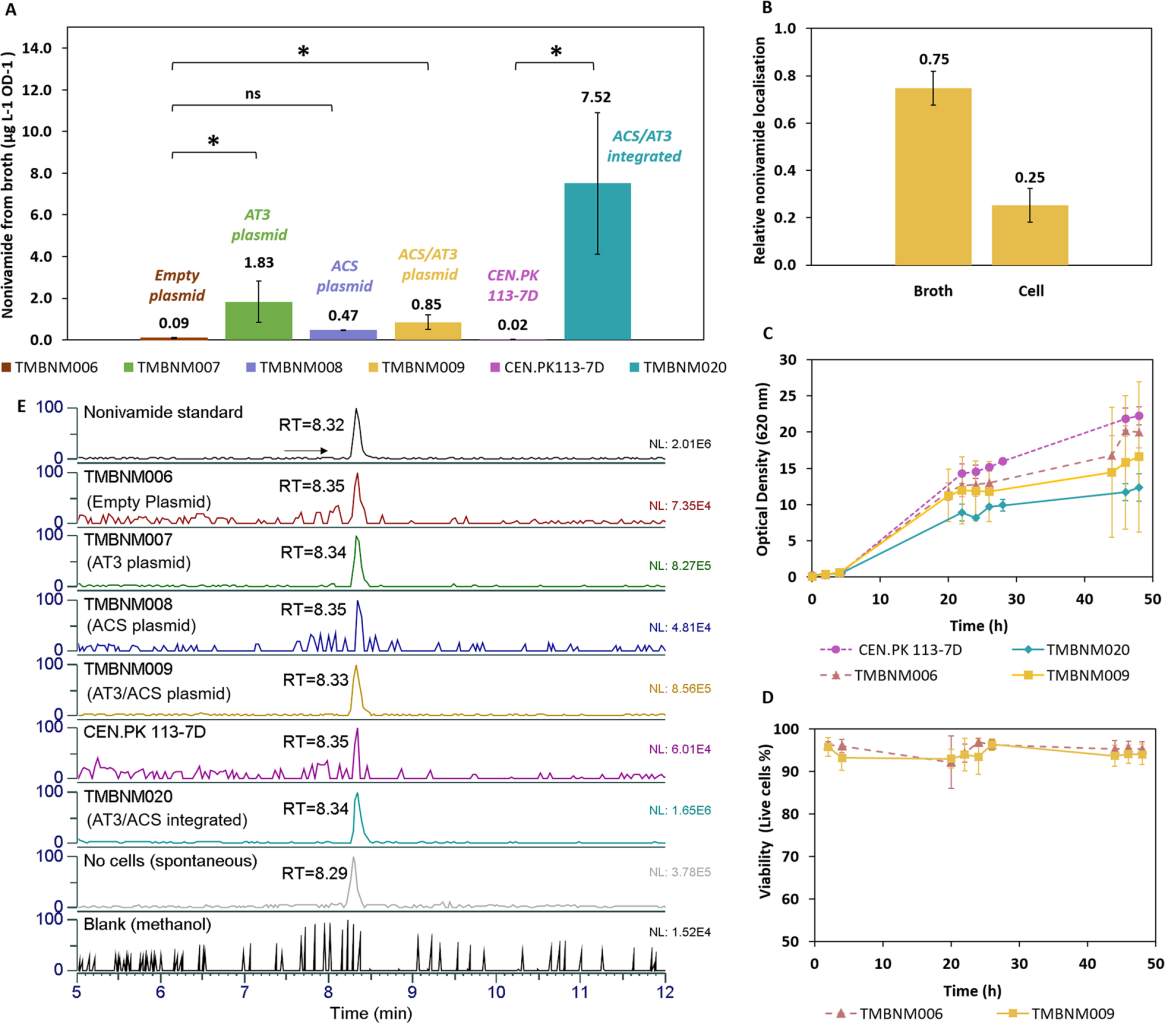
Whole-cell production of nonivamide. **A** Nonivamide amounts (µg L^−1^ OD^−1^) detected in the media broth fraction, normalized per OD_620_. Student t-test was done for significance between the values (p > 0.05 not significant (ns), p < 0.05 significant (*). **B** Relative nonivamide localization of the extracted product in broth and cell fraction from the production strain TMBNM009 (AT3/ACS plasmid). **C** Optical density at 620 nm measured throughout the cultivation in bioreactors of CEN.PK 113-7D, TMBNM020 (AT3/ACS integrated), TMBNM006 (empty plasmid) and TMBNM009 (AT3/ACS plasmid). **D** Viability of TMBNM006 (empty plasmid) and TMBNM009 (AT3/ACS plasmid) cells throughout the cultivation in bioreactors, assayed by flow cytometry. **E** Representative graphs from LC-MS analysis for confirming nonivamide presence in broth fraction (Ion extracted ESI+ mass range m/z = 294.15–294.25, nonivamide standard RT = 8.32 min). Observe that the scale differs between samples and is normalized to the level of the highest peak (NL = Normalization level)

Constructed over-expression strain TMBNM009 (plasmid AT3/ACS) and TMBNM020 (integrated AT3/ACS), as well as their control strains, TMBNM006 (empty plasmid (EP)) and CEN.PK113-7D, respectively, were cultivated in defined mineral medium in bioreactors. Precursors for nonivamide (vanillylamine and nonanoic acid) were added at low amounts together with glucose allowing for cell growth to occur, thereby ensuring the availability of CoA-SH and ATP. After 48 h of cultivation, broths and cell pellets were extracted separately, and nonivamide was analysed by HPLC and LC-MS/MS (Fig. 3A, E). TMBNM020 (integrated AT3/ACS) produced nonivamide at a level of 7.52 ± 3.40 µg L^−1^ OD^−1^ in the broth fraction, and TMBNM009 (plasmid AT3/ACS) produced at a level of 0.85 ± 0.35 µg L^−1^ OD^−1^ (Fig. 3A). Nonivamide was also detected in the cell pellet fractions, but to a lower level than in the broths. The relative localisation of nonivamide separated in broth and in cell pellet was 0.75 ± 0.07 and 0.25 ± 0.07, respectively (Fig. 3B). Product yield (Yp/s) was 0.24 ± 0.07 mg nonivamide/g nonanoic acid for TMBNM009 (plasmid AT3/ACS) and 0.96 ± 0.50 mg nonivamide/g nonanoic acid TMBNM020 (integrated AT3/ACS). The low product yields could probably be explained by low activity of the recombinant NAT and/or CL enzymes. The strain with chromosomal integration of *AT3* and *ACS* genes (TMBNM020), showed higher mean production, however the difference was not statistically significant (p > 0.1) compared to the plasmid strain (TMBNM009). Protein analysis by LC-MS/MS, focusing on the heterologously expressed proteins, confirmed the presence of the ACS and AT3 proteins in both the multicopy (TMBNM009) and single copy strain (TMBNM020). ACS was detected and well covered with 32 peptides, with the N-terminal peptide and a peptide close to the C-terminal found. The AT3 protein was also detected and confirmed to be present however the coverage was lower with 11 peptides (Additional file 1: Table S1).

Remarkably, a small amount of nonivamide was observed also for the control strains. The production was however significantly lower than the production strains, but still within the detection limit of the HPLC. The nonivamide level for TMBNM020 (integrated AT3/ACS) was around 380-fold higher than the level obtained for the control strain CEN.PK 113-7D. Additionally, the plasmid AT3/ACS strain TMBNM009, when compared to its control with an empty plasmid (TMBNM006), produced around 10-fold more nonivamide. To examine if the reaction occurs spontaneously in the bioreactor setup, 500 mL minimal medium supplemented with substrates was incubated for 48 h at 30 °C without any cells. The broth was extracted with ethyl acetate, which was then evaporated, and the remaining fraction was analysed with LC-MS/MS (Fig. 3E). A low amount was observed (3.73 ± 0.71 µg L^−1^), although still above the detection limit. This indicates that the level of nonivamide produced with the control strains TMBNM006 (2.49 ± 0.96 µg L^−1^) and CEN.PK 113-7D (0.36 ± 0.50 µg L^−1^) could be due to spontaneous formation. However, the amount in TMBNM009 (23.89 ± 6.81 µg L^−1^) and TMBNM020 (96.08 ± 56.28 µg L^−1^) was significantly higher. Therefore, nonivamide is unlikely to only come from spontaneous production, demonstrating the amide-forming activity of AT3 and ACS in yeast.

In order to investigate the activity of yeast-native acyl-CoA synthetases towards nonanoic acid, a strain expressing only *AT3* was constructed [TMBNM007 (plasmid AT3)]. Indeed, after 48 h cultivation of this strain in medium supplemented with precursors, 1.83 ± 0.99 µg L^−1^ OD^−1^ nonivamide was observed (Fig. 3A, E). The amount detected for TMBNM007 (plasmid AT3) was significantly higher than the nonivamide produced in the broth by TMBNM006 (empty plasmid) (p = 0.02). Since AT3 requires nonanoyl-CoA as substrate, this indicates that baker’s yeast carries some endogenous CL activity towards nonanoic acid.

To investigate if yeast also carried any promiscuous capsaicin synthase activity from endogenous acyltransferases, a strain only expressing *ACS* coding for the acyl-CoA ligase was constructed [TMBNM008 (plasmid ACS)]. After 48 h batch cultivation, nonivamide was detected at a concentration of 0.47 ± 0.02 µg L^−1^ OD^−1^ (Fig. 3A, E), which was about twice the amount measured from the spontaneous formation without cells described above. Significantly more (p = 0.02) nonivamide was detected in the TMBNM007 carrying the acyltransferase AT3, compared to the product quantified from TMBNM008 (plasmid ACS). Additionally, the difference in nonivamide amount between the control TMBNM006 (empty plasmid) and TMBNM008 (plasmid ACS) was not significantly different (p = 0.12), which confirms the need for the heterologous AT3 enzyme in order to produce higher levels of nonivamide in yeast.

Yeast growth was negatively affected in cultures where nonivamide was produced (Fig. 3C). After 48 h, the final biomass measured as optical density was 20.3 ± 1.6 and 14.3 ± 8.4 OD_620_ for TMBNM006 (empty plasmid) and TMBNM009 (plasmid AT3/ACS), respectively. For CEN.PK 113-7D and TMBNM020 (integrated AT3/ACS), final biomass measured as optical density was 22.3 ± 1.3 and 12.4 ± 1.9 OD_620_, respectively. Final biomass was 30–40% lower in production cultures. There were no growth impairments in absence of precursors (data not shown), demonstrating that it was indeed the production of nonivamide that gave the negative impact and not the burden of heterologous gene expression. The concentration of nonivamide produced was lower (0.09–0.32 µM) than what was found to be inhibitory after external supplementation (Fig. 3B) Nonivamide was not reached at a sufficient level to negatively affect cell viability, which remained above 92% (Fig. 3D).

### Screening of *N*-acyltransferase and CoA-ligase enzymes

In pursuit of more efficient NATs and CLs, several enzymes from plant and bacterial origin with previously shown amide-forming activity on relevant substrates [27], were explored as candidates for capsaicinoid formation. Philpott et al. [27] systematically evaluated 9 CLs and 45 NATs separately for CL activity on various carboxylic acids and NAT activity towards a range of CoA esters and amines, as well as in combination for amide forming activity. They identified several CLs with activity towards aliphatic carboxylic acids, namely PhCL (4-coumarate: CoA ligase from *Petunia hybrida*) and IpfF (Ibuprofen CoA ligase from *Sphingomonas* sp.* Ibu-2*). Additionally, several NAT enzymes were determined to have activity towards various amides, especiallyaromatic amines (4-(aminomethyl)benzonitrile), and various thioesters, especially aliphatic CoA-esters. The promiscuity of these enzymes could allow for flexibility in the amide bond formation. The NATs were: PaAT (Arylamine *N*-acetyltransferase from *Pseudomonas aeruginosa*), CaAT (Tyramine *N*-(hydroxycinnamoyl) transferase from *C. annuum*) and SlAT (*N*-hydroxycinnamoyl-CoA:tyramine *N*-(hydroxycinnamoyl) transferase THT1-3 from *Solanum lycopersicum*). In this study, this panel of NAT and CL enzymes, including ACS and AT3, was introduced in yeast in varying combinations.

**Fig. 4:**
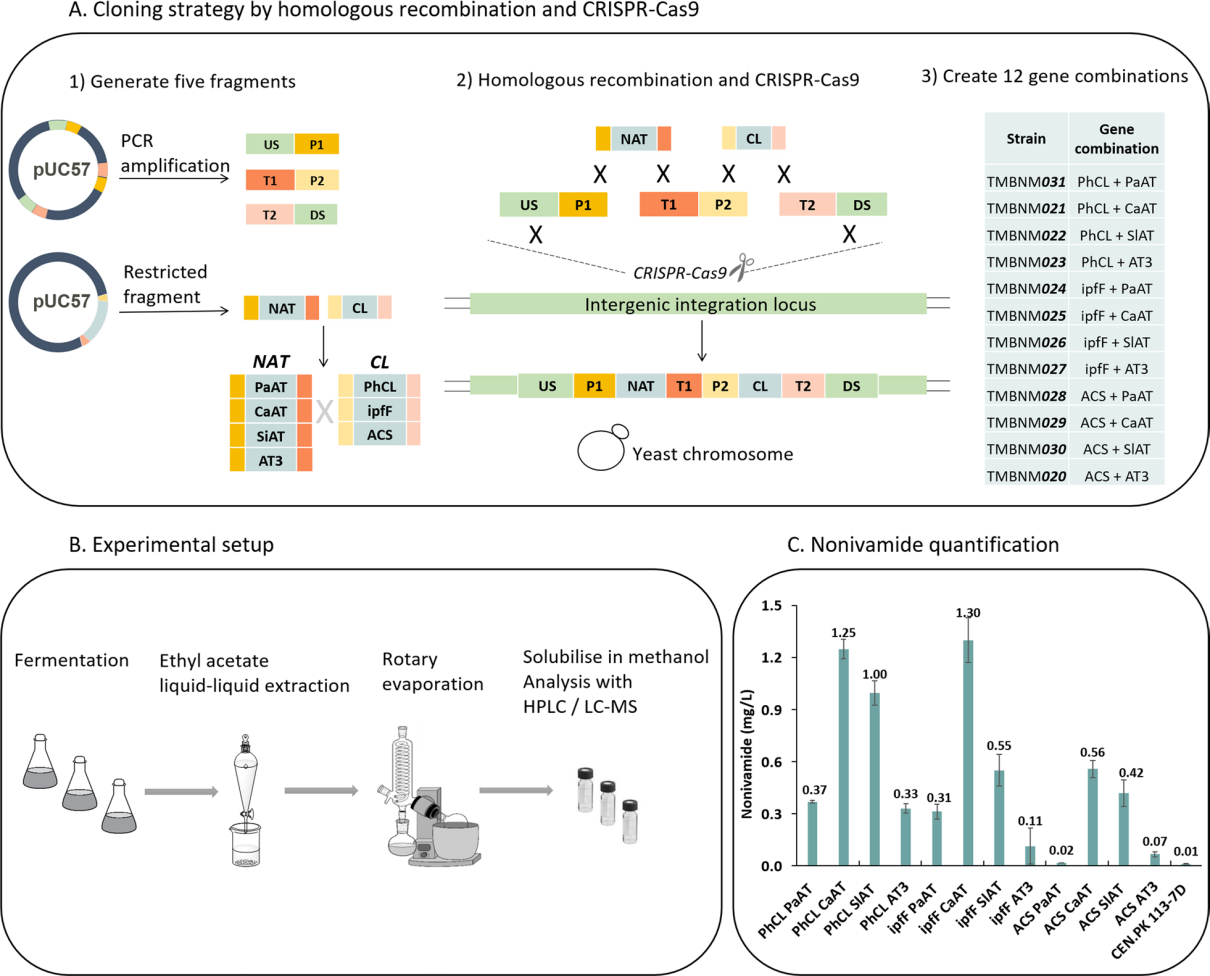
Combinatorial design study for screening NAT and CL enzymes. **A** Overview of the cloning strategy for generating a selection of twelve strains expressing different combinations of NAT and CL enzymes. Five fragments were generated by PCR and restriction enzymes, with homologous overlaps allowing for homologous recombination. CRISPR-Cas9 toolbox was used to make a double stranded break in an intergenic integration locus, and the fragments were used to repair the break, leading to their integration in the yeast chromosome. *US *upstream region of the integration locus, *DS *downstream region of the integration locus, *P1/P2 *promotor 1 and 2, *T1/T2 *terminator 1 and 2, **B** experimental flow for the screening of strains for nonivamide production. **C** HPLC analysis of nonivamide detected and quantified (mg L^−1^) for each strain

To enable efficient chromosomal integration of both genes a new modular cloning approach was developed. The method combines a previously constructed CRISPR-Cas9 toolbox [38] with an herein developed strategy for targeted combinatorial assembly of multiple fragments by homologous recombination (Fig. 4A). First, the genes coding for the panel of enzymes were codon-optimized, synthesized and cloned on a plasmid flanked by overlapping homologous regions to a promotor and a terminator on each end of the gene sequences. These sequences were then cut from the plasmid using restriction enzymes. PCR was used to obtain three other fragments coding for: (i) upstream homologous region and a promotor, (ii) terminator and a second promotor and (iii) second terminator and a downstream homologous region (Fig. 4A). A double-strand break (DSB) was introduced in the XI-5 chromosomal region by Cas9 directed by a previously developed gRNA [38]. The five fragments were introduced as donor DNA in the transformation mixture (at equal proportions), and consequently the DSB was repaired by homologous recombination of the fragments. The transformation efficiency varied between 35 and 600 CFU/mL depending on the gene combinations transformed, nonetheless, 60–100% of tested colonies from the plate were confirmed to have correct integration by colony PCR. The method was shown to be efficient and relatively simple for the combinatorial gene assembly in this work.

The library of strains, including an unmodified control (CEN.PK 113-7D), were screened for nonivamide production in a 50 mL shake flask cultivation (Fig. 4B) for 48 h. Nonivamide was produced in all strains in different levels, demonstrating that both NAT and CL influenced the level of nonivamide reached (Fig. 4C). Looking at the enzymes individually, the CaAT enzyme was shown to be the most successful NAT in yeast for the formation of nonivamide in all combinations with any CL enzyme. The CL with the individually highest activity was PhCL, closely followed by IpfF. The highest titres were 1.25 ± 0.06 mg L^−1^ for TMBNM021 (PhCL/CaAT) and 1.30 ± 0.13 mg L^−1^ for TMBNM025 (IpfF/CaAT). The yield (Yp/s) was 12.49 ± 0.56 mg nonivamide/g nonanoic acid for TMBNM021 (PhCL/CaAT) and 13.00 ± 1.29 mg nonivamide/g nonanoic acid for TMBNM025 (IpfF/CaAT). TMBNM025 showed around 20-fold increase in nonivamide in comparison to TMBNM020 (ACS/AT3), and around 100-fold increase than the unmodified laboratory strain CEN.PK 113-7D.

### Evaluation of TMBNM025 for whole-cell biosynthesis of nonivamide in a bench-scale bioreactor

**Fig. 5:**
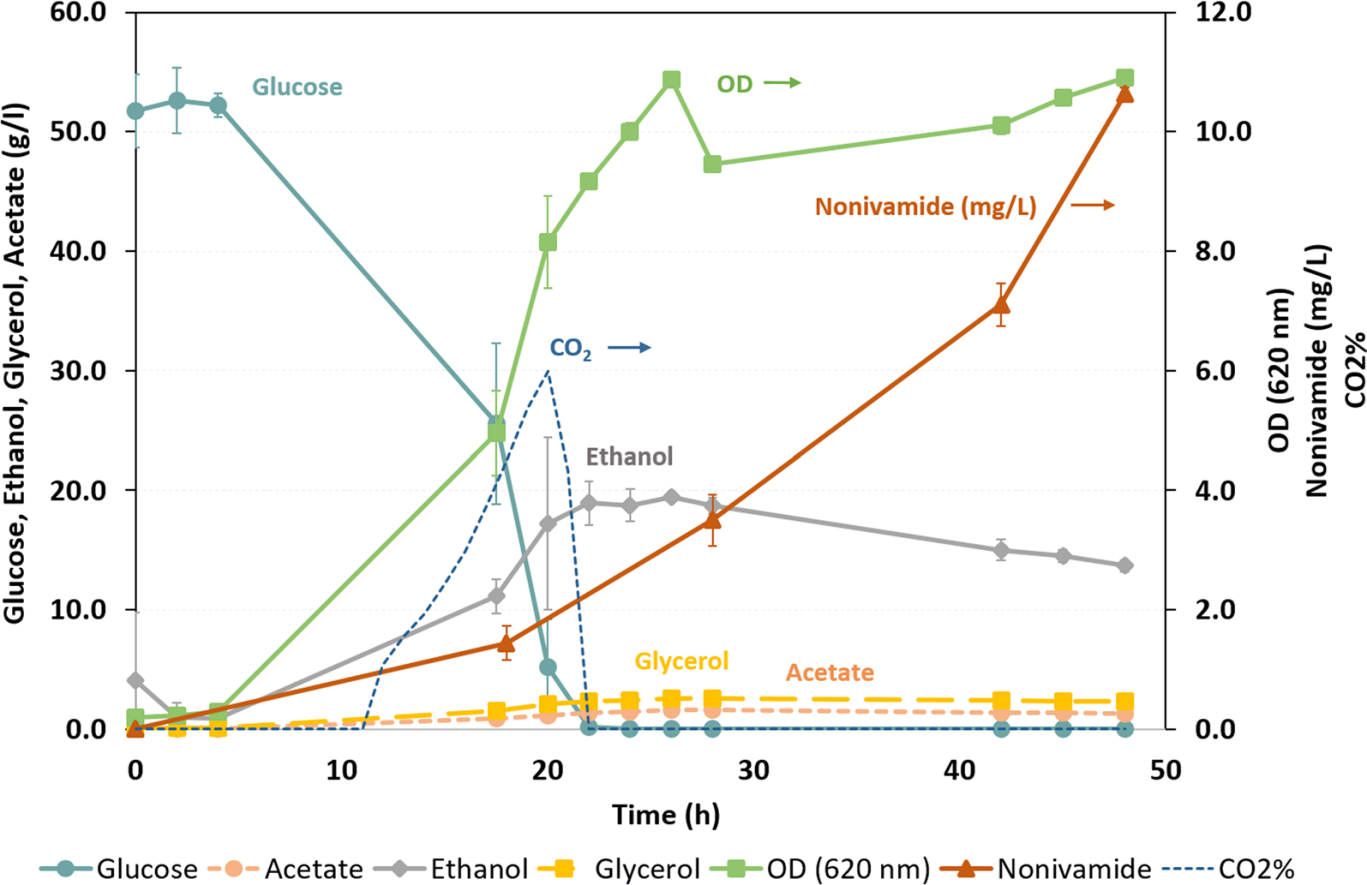
Aerobic batch cultivation of TMBNM025 (IpfF/CaAT). Glucose (g L^−1^), ethanol (g L^−1^), acetate (g L^−1^) and glycerol (g L^−1^) are shown on the left axis, while OD (620 nm) and nonivamide (mg L^−1^) and CO_2_% are shown on the right axis. Nonivamide is produced throughout the whole cultivation with 10.6 ± 0.1 mg L^−1^ as the highest amount detected at 48 h

The best performing strain TMBNM025 (IpfF/CaAT) for nonivamide production was further characterised in a well-controlled fermenter setup in defined medium supplemented with vanillylamine and nonanoic acid. The strain showed diauxic growth (Fig. 5), although after glucose depletion at 24 h only a slight growth on ethanol was observed, indicating a low respiratory capacity under the applied aeration conditions. The maximum ethanol concentration reached was 19.5 ± 0.4 g L^−1^ after which it was slowly consumed throughout the rest of the cultivation. Small amounts of glycerol and acetate were also produced (< 2.6 g L^−1^). Nonivamide was produced throughout the entire cultivation and reached a final concentration of 10.6 ± 0.1 mg L^−1^, with a product yield (Yp/s) from nonanoic acid of 106 ± 1.0 mg nonivamide/g nonanoic acid (molar yield of 5.7 ± 0.1%).

## Discussion

Yeast could become a useful production platform for capsaicinoids. Here, specific NAT and CL enzymes, catalysing the amide-forming step in the route towards capsaicinoids, were identified and used to establish yeast-based production of nonivamide at mg-scale. The developed yeast system holds substantial potential to also be used for synthesis of a wide range of other capsaicinoids by supplementing the reaction broth with specific precursors. The developed method for combinatorial cloning of NATs and CLs, and the analytical pipeline for evaluating the production of specific capsaicinoids may be useful for identifying enzymes for the production of novel unnatural capsaicinoids. NAT and CL enzymes are present in many organisms for biosynthesis of secondary metabolites, and that natural variability may be harnessed for synthesis of various amides.

The most effective individual enzyme for production of nonivamide from the NATs investigated here was CaAT, a tyramine *N*-hydroxycinnamoyl transferase from *C. annuum*. The enzyme has already been shown to accept a relatively broad substrate range of different amines and acyl-CoAs [27]; however it has not been found to carry capsaicinoid synthase activity. Previously the CaAT enzyme has been purified and characterized in vitro for amide synthesis from *N*-hydroxycinnamic acid and tyramine, feroloyltyramine and *p*-coumaroyltyramine [39]. Here, it can be concluded that CaAT also has high activity in yeast and can be used for whole-cell production of nonivamide. BLAST [40] search of plant sequences showed that the protein is present in several chili pepper species (such as *C. annum*, *C. chinense*, and *Capsicum baccatum*) with over 99% protein sequence identity. CaAT may thus have an important but overlooked catalytic role for the amide-forming step in the capsaicinoid pathway in chili pepper plants. Capsaicinoid synthase activity in chili is credited to the AT3 enzyme, which has been described as key for capsaicinoid biosynthesis [36]. Nonivamide was indeed detected in yeast strains expressing the AT3 enzyme, however the production was around 4 to 12-fold lower compared to yeast expressing CaAT depending on which CL partner was used. It is unclear whether the lower activity for AT3 is due to weak expression in yeast, and if it also carries lower specific activity in plantae. Several plant enzymes involved in biosynthesis of natural products have previously been found to contain a chloroplast transfer peptide (cTP), which is cleaved off during the formation of the mature protein. Analysis of the protein sequences with the subcellular localisation prediction tool TargetP 2.0 [41] showed a higher likelihood for the presence of an *N*-terminal cTP in AT3 and not in CaAT. The presence of a plastid targeting sequence may influence enzyme localisation and/or activity in yeast, similarly to what was previously found for a recombinant limonene synthase [42, 43]. Biochemical characterisation of both enzymes in purified form could in the future shed light into the specific enzyme activity as well as their potential structural differences. SlAT, a hydroxycinnamoyl-CoA:tyramine *N*-(hydroxycinnamoyl) transferase THT1-3 from tomato (*Solanum lycopersicum*), was also found here to carry capsaicinoid synthase activity in yeast. There are several THT homologous enzymes in tomato, previously characterized for synthesis of *N*-(hydroxycinnamoyl)-amines, compounds presumed to be involved in cell-wall strengthening and plant defence against pathogens. [44]. They are known to be present in potato, tobacco, as well as in several chili pepper species with over 84% protein sequence identity to SlAT after a BLAST [40] search. A purified THT enzyme from *C. annuum* was shown to have activity over several cinnamoyl-CoA esters with tyramine [45]. These enzymes also have been shown to have a broad substrate range for synthesis of aromatic amides [27], as also seen from this work.

The most efficient CLs were PhCL, a 4-coumarate: CoA ligase 1 from *Petunia hybrida*, and IpfF, an Ibuprofen CoA ligase from *Sphingomonas* sp.* Ibu-2*. Both enzymes displayed higher activity for the activation of the C9:0 free fatty acid in yeast than the ACS, which was previously annotated for CoA-activation of medium chain fatty acids in chili pepper. PhCL has previously been used for catalyzing formation of CoA thioesters of benzenoid derivatives such as 4-coumaric, caffeic, ferulic, trans-cinnamic and benzoic acid [46]. Similarly, IpfF, was previously characterised as CL acting on aromatics such as ibuprofen [47]. Here it is found that both can be active towards aliphatic carboxylic acids as well. The application of these CLs together with NATs with broad substrate range, such as SlAT and CaAT, may be useful for production of a wide range of unnatural capsacinoids with variation in the aromatic moiety as well as in the aliphatic side chain. It could be speculated that the developed yeast system may be used for generation of compound libraries of capsaicinoids by whole-cell biocatalysis, and could be screened for novel bioactivities such as TRPV1 modulating activity.

The presence of promiscuous native yeast enzymes possibly acting on the substrates could contribute to nonivamide formation. Alcohol acetyltransferase enzymes Atf1, Atf2, Eeb1 and Eht1 are involved in ester synthesis in yeast [48–50], and they could be hypothesised to carry low activity for amide formation. However, yeast was found not to carry substantial native capsaicinoid synthase background activity. Nonanoic acid, on the other hand, was converted to nonanoyl-CoA in yeast. This is evidenced by the formation of a significant amount of nonivamide in the strain only expressing the AT3 enzyme, thus relying on native CoA-ligase activity for activation of nonanoic acid to nonanoyl-CoA used in the reaction. In *S. cerevisiae*, the two main acyl-CoA synthetases Faa1 and Faa4 activate long fatty acids in the cytoplasm before the Pxa1p-Pxa2p protein complex, belonging to ABC transporters, carries them to the peroxisome [51]. However, they have not previously been reported to carry the desired activity towards nonanoic acid. *FAA2* has been characterised to code for a medium chain fatty-acyl-CoA synthetase (Faa2p) localised in the peroxisome, where fatty acid β-oxidation takes place [52]. Whether Faa2p could also be found in the cytoplasm, where it could carry some activity against nonanoic acid is unclear.

Synthesis of capsaicinoids in the plant takes place in the fruit, in the epidermal cells of the placenta, more precisely within the endoplasmic reticulum, from where it is secreted in cell vesicles, and is present in large part in the vacuole. During ripening, they are translocated towards the cell membrane and the surface of the cell cuticle and accumulate in a subcuticular cavity forming blisters [53, 54]. In yeast, nonivamide was found to be partly associated with the cell pellet, which may not be surprising since the product is practically insoluble in aqueous solution and would therefore be likely to bind non-covalently to more hydrophobic cell constituents. Similar behaviour of interaction with the cell membrane has been observed previously by monitoring the intrinsic fluorescence of capsaicin in a synthetic lipid bilayer membrane [34]. It can be speculated that this caused the observed growth impairment, possibly from a resulting suboptimal membrane fluidity or integrity. Whether product localisation could be steered by introducing a specific transport protein resulting in increased efflux, or if the association to cell pellet is inevitable due to hydrophobic interactions to the cell envelope is still unclear.

Another cause for the low conversion may be low availability of substrates caused by poor transport from the extracellular surrounding. Vanillylamine is unlikely the limiting factor, since it was supplemented in 4-fold excess to nonanoic acid. Furthermore, native yeast lacks vanillin transaminase activity [13, 14], and no conversion of vanillylamine to vanillin was observed. Nonanoic acid has low water solubility, sticks on surfaces of the bioreactor and is likely to accumulate in the cell membrane and other lipophilic cell constituents. The inhibition mechanism of capsaicinoids may be similar to medium chain fatty acids, as described previously [35]. The pKa of the investigated SFAs were all in the range between 4.82 and 5.30 [55], resulting in 94–98% ionization at culture pH used (pH 6.5), and is therefore not the determining factor for the higher inhibition observed for medium-chain FAs. Instead, lipophilicity, which determines the partitioning of the compound between hydrophilic and hydrophobic cell constituents, may explain the difference between short and medium-chain length FA, where the latter are more prone to cause damage to the plasma membrane. However, the low inhibition of long-chain FA, with highest lipophilicity, does not apply to this logic. Long-chain FA are common in yeast, consisting mostly of C16 and C18 phospholipids [56], and is thus likely not to affect the membrane properties to the same extent as the non-native medium-chain FAs. Altogether, this indicates that *S. cerevisiae* might be a more suitable host for production of capsaicinoids with shorter or long acyl chains. Process engineering solutions such as substrate feeding at low concentrations in a fed-batch setup, combined with in situ product removal could counteract the toxic effects of substrate or product. For example, addition of oleic acid (C18:1) was previously shown to decrease the inhibitory effect of the medium-chain fatty acid octanoic acid [35], and may enable an increased substrate load. De novo synthesis in the cell, as shown previously for medium-chain fatty acids [12], may be another approach to improve the reaction by balancing inhibitory substrate levels, as well as to relieve issues with intermembrane transport as potential bottleneck. Recently, fungal type I fatty acid synthase was engineered for in vivo production of short and medium chain fatty acids by the expression of heterologous genes [12, 57]. Learnings from this may be applied to produce specific medium-chain fatty acids in vivo at a level in tune with the activity of the NAT-CL cascade, thereby reducing build-up of the inhibitory fatty acids.

## Conclusions

In this study, an active biosynthetic pathway for capsaicinoids was constructed in *S. cerevisiae* and utilized for whole-cell biocatalytic production of 10.6 mg L^−1^ nonivamide from supplemented precursors in well-controlled bench-scale fermentors. We demonstrated that CaAT from *C. annuum* was a significantly more efficient capsaicinoid synthase in yeast than AT3 (or Pun1), which is typically credited for capsaicinoid biosynthesis in *Capsicum* spp. We also identified SlAT from tomato to efficiently catalyse the formation of nonivamide in yeast, however to a slightly lower level than CaAT. The CLs IpfF and PhCL were efficient partners of the NAT enzymes, and were able to produce nonanoyl-CoA in yeast from supplemented free fatty acid. In future work, challenges for the complete biosynthesis of capsaicinoids in yeast could be addressed by combining previously developed de novo routes to vanillin and specific free fatty acids.

## Methods

### Chemicals, strains and media

All chemicals were purchased from Sigma-Aldrich (or Merck; USA), except for vanillylamine that was purchased from Alfa Aesar (Massachusetts, United States) and C3:0, C4:0 saturated fatty acids, which were from AppliChem (Darmstadt, Germany).

Strains and plasmids are listed in Table 1. *Escherichia coli* DH5α was used for subcloning. The leucine auxotrophic strain *S. cerevisiae* strain CEN.PK 113-16B and the prototrophic CEN.PK 113-7D were used for recombinant gene expression. The prototrophic CEN.PK 113-7D was also used for the substrate inhibition assays. Strains were stored at − 80 °C in 25% (v/v) glycerol. *E. coli* transformants were selected on Lysogeny Broth (LB) medium (10 g L^−1^ tryptone, 5 g L^−1^ yeast extract, 10 g L^−1^ NaCl; 15 g L^−1^ agar for plates) supplemented with 100 µg/mL ampicillin (IBI Shelton Scientific, CT, USA) and grown at 37 °C. *S. cerevisiae* transformants were selected on Yeast Nitrogen Base (YNB) medium without amino acids (6.7 g L^−1^ yeast nitrogen base without amino acids, 20 g L^−1^ glucose, 20 g L^−1^ agar-agar for plates, buffered with 0.1 M potassium phosphate buffer). For propagation of CEN.PK 113-16B, leucine was added to the YNB medium in recommended concentrations of 500 mg L^−1^ [58]. CEN.PK 113-7D was maintained on a plate with Yeast Peptone Dextrose (YPD) medium (20 g L^−1^ tryptone, 10 g L^−1^ yeast extract, 20 g L^−1^ glucose; 20 g L^−1^ agar–agar for plates). For the bioconversion, defined mineral medium [59] buffered at pH 6.5, and supplemented with capsaicinoid precursors was used.

**Table 1:** Strains and plasmids

	Description	References
*E. coli* strain		
DH5α	*E. coli*; electro competent cells	Life Tech.
DH5α Pun 1	*E. coli; yEP181-AT3*	This study
DH5α ACS	*E. coli; yEP181-ACS*	This study
DH5α ACS-AT3	*E. coli; yEP181- ACS, AT3*	This study
*S. cerevisiae* strain		
CEN.PK 113-7D	MATα; prototrophic strain	Euroscarf.
CEN.PK 113-16B	MATα; leu2	Euroscarf.
TMBNM006	CEN.PK 113-16B; *leu2:yEP181*	This study
TMBNM007	CEN.PK 113-16B; *leu2::yEP181::AT31*	This study
TMBNM008	CEN.PK 113-16B; *leu2::yEP181::ACS*	This study
TMBNM009	CEN.PK 113-16B; *leu2::yEP181::AT3, ACS*	This study
TMBNM020	CEN.PK 113-7D; XII-5:: *TEF1*p-*ACS*-*PGK1*t; *GDP*p-*AT3*-*ADH1*	This study
TMBNM021	CEN.PK 113-7D; XII-5:: *TEF1*p-*PhCL*-*PGK1*t; *GDP*p-*CaAT*-*ADH1*	This study
TMBNM022	CEN.PK 113-7D; XII-5:: *TEF1*p-*PhCL*-*PGK1*t; *GDP*p-*SlAT*-*ADH1*	This study
TMBNM023	CEN.PK 113-7D; XII-5:: *TEF1*p-*PhCL*-*PGK1*t; *GDP*p-*AT3*-*ADH1*	This study
TMBNM024	CEN.PK 113-7D; XII-5:: *TEF1*p-*IpfF*-*PGK1*t; *GDP*p-*PaAT*-*ADH1*	This study
TMBNM025	CEN.PK 113-7D; XII-5:: *TEF1*p-*IpfF*-*PGK1*t; *GDP*p-*CaAT*-*ADH1*	This study
TMBNM026	CEN.PK 113-7D; XII-5:: *TEF1*p-*IpfF*-*PGK1*t; *GDP*p-*SlAT*-*ADH1*	This study
TMBNM027	CEN.PK 113-7D; XII-5:: *TEF1*p-*IpfF*-*PGK1*t; *GDP*p-*AT3*-*ADH1*	This study
TMB NM 028	CEN.PK 113-7D; XII-5:: *TEF1*p-*ACS*-*PGK1*t; *GDP*p-*PaAT*-*ADH1*	This study
TMB NM 029	CEN.PK 113-7D; XII-5:: *TEF1*p-*ACS*-*PGK1*t; *GDP*p-*CaAT*-*ADH1*	This study
TMB NM 030	CEN.PK 113-7D; XII-5:: *TEF1*p-*ACS*-*PGK1*t; *GDP*p-*SlAT*-*ADH1*	This study
TMB NM 031	CEN.PK 113-7D; XII-5:: *TEF1*p-*PhCL*-*PGK1*t; *GDP*p-*PaAT*-*ADH1*	This study
Plasmid		
yEP181lac	Yeast episomal plasmid, 2µ	[60]
pCfB3050	gRNA sequence for targeting chromosomal site XII-5	38
pNM001	pUC57; AmpR; *TEF1*p-*ACS*-*PGK1*t; *GDP*p-*AT3*-*ADH1*t	Genescript
pNM002	y*EP181*::*AT3*	This study
pNM003	y*EP181*::*ACS*	This study
pNM004	y*EP181*::*ACS, AT3*	This study
pNM005	pUC57mini::PaAT, AmpR	Genescript
pNM006	pUC57mini::CaAT, AmpR	Genescript
pNM007	pUC57mini::SlAT, AmpR	Genescript
pNM008	pUC57mini::PhCL, AmpR	Genescript
pNM009	pUC57mini::IpfF, AmpR	Genescript

### Molecular biology methods and plasmid construction

For the molecular biology, standard methods were used according to Sambrook and Russell [60]. DreamTaq DNA polymerase and Phusion polymerase (Thermo Scientific, Waltham, MA, US) were used for PCR and cloning, respectively. Primers used for colony PCR were from Eurofins MWG Operon (Ebersberg, Germany). PCR products were purified using the PCR purification kit (Thermo Scientific, Waltham, MA, US). Size analysis of DNA fragments was done using electrophoresis in agarose gel (0.8%) with MUPID-exU submarine electrophoresis system (Mupid; Tokyo, Japan).

Plasmids were designed using SnapGene software (GSL Biotech LLC, Version 3.0.3.) and purchased from Genescript (Piscataway, NJ, USA). The pNM001 was constructed to allow for subsequent cloning of individual genes into the yeast multicopy plasmid yEP181lac [61]. Initially, the pNM001 and the episomal plasmid yEP181lac were digested with FastDigest restriction enzymes (Thermo Scientific, Waltham, MA, US). The digestion of the pNM001 was followed by ligation into, also digested, yEP181lac, using T4 ligase (Thermo Scientific, Waltham, MA, US), to produce pNM004 plasmid. To create the pNM003 plasmid, the AT3 fragment was digested from the pNM003 and the plasmid was self-ligated. The same was done to create the pNM002 plasmid, where the ACS fragment was digested from the pNM003 and the plasmid was self-ligated. For the chromosomal integration of the ACS-AT3 cassette, as well as the different NAT-CL gene combinations, backbone plasmid and gRNA was used from the CRISPR-cas9 system of Jessop-Fabre et al. [38]. The plasmids pNM005-009 were designed to have 50 bp homologous regions to the chromosomal integration site XI-5, before the beginning of the promotor region and the end of terminator region. All the gene sequences were codon optimised for expression in *S. cerevisiae* (Additional file 1). All of the plasmids were verified using restriction enzyme analysis and/or Sanger sequencing according to the Eurofins Submission Guide (Eurofins Genomics, Ebersberg, Germany).

### Strain construction and verification

All plasmids were first propagated in *E. coli* DH5α competent cells according to the Inoue protocol [62] and purified using GeneJet plasmid MiniPrep kit (Thermo Scientific, Waltham, MA, US). Subsequently, pNM002, pNM003 and pNM004 were transformed in the leucine auxotrophic *S. cerevisiae* strain CEN.PK. 113-16B according to the lithium acetate protocol [63] with the modification of including DMSO (10% v/v) as a step to increase the permeability of the cells before heat-shock [64], generating strains TMBNM007, TMBNM008 and TMBNM009. A strain containing an empty yEP181lac plasmid was constructed as a negative control for the experiments, named TMBNM006. For the chromosomal integration of the ACS-AT3 cassette, the backbone plasmid, carrying the ACS-CS cassette, was digested and transformed in the prototrophic *S. cerevisiae* strain CEN.PK. 113-7D, already containing the Cas9 plasmid, together with the compatible gRNA plasmid according to the same lithium acetate protocol mentioned above. Selected colonies were verified with PCR in order to confirm that a correct integration occurred.

For the construction of the NAT-CL combinatorial library, the same CEN.PK. 113-7D, already containing the Cas9 plasmid was used. The gRNA plasmid for guiding the double stranded brake in the XI-5 chromosomal region was used. Gene sequences with the 50 bp overlap sequence from pNM005-009 were restricted from the plasmid using restriction enzymes. Additionally, three other fragments from pNM001 were PCR amplified. Finally, the donor DNA contained 5 fragments coding for: (i) XI-5 upstream homologous region and a promotor, (ii) a NAT coding gene, (iii) a terminator and a second promotor, (iv) a CL coding sequence and (v) second terminator and a XI-5 downstream homologous region. These fragments were used to repair the double-stranded break resulting in the chromosomal insertion of the five fragments. Successful verification with PCR was performed both inside and outside of the inserted region in order to confirm the proper integration.

The expression of AT3 and ACS was confirmed and their levels determined with protein LC-MS/MS. Overnight pre-culture from TMBNM009 and TMBNM020 was prepared from a single colony and used for inoculating two 250 mL shake flasks with 25 mL Verduyn minimal media starting with OD ~ 1. After 8 h cells were collected, washed twice with PBS, pelleted and cell pellets were snap frozen in liquid nitrogen and stored at − 80 °C until sample analysis. Samples were digested with trypsin and analysed on a quadrupole LC-MS/MS instrument at the Department of Immunotechnology, Faculty of engineering, Lund University, Sweden.

### Shake-flask cultivations

The *S. cerevisiae* strains were cultivated at 30 °C under shaking at 180 rpm, in a defined mineral medium with 20 g L^−1^ glucose and buffered at pH 6.5 without addition of precursors. Overnight pre-culture was inoculated from a single colony and shake flask was inoculated with an initial OD620 nm of 0.5 in 25 mL or 50 mL depending on the experiment. Cell growth was monitored by measuring optical density at 620 nm (OD620) with a spectrophotometer (Ultrospec 2100 pro UV/Visible spectrophotometer, Amersham Biosciences, Buckinghamshire, United Kingdom).

### Growth inhibition assays

Growth inhibition by capsaicin, nonivamide, vanillylamine and saturated fatty acids (C3:0, C4:0, C6:0, C8:0, C9:0, C10:0, C12:0, C16:0) was assayed using CEN.PK113-7D. A pre-culture was prepared by inoculating a single colony in 5 mL YNB medium and was incubated overnight under shaking at 30 °C. The pre-culture was used to inoculate a 96-well microtiter plate (Sarstedt, Germany) containing YNB medium supplemented with varying concentrations (0–2 mM; 0–13 mM) of compounds at a starting OD at 620 nm of 0.1 in a final volume of 250 µL. Due to low solubility in water, except for vanillylamine, compounds were first solubilised in 99,5% ethanol and supplemented at an equal volume [9.5% (v/v)]. A Multiskan™ FC Microplate Photometer (Thermo Fisher Scientific, USA) plate reader was used for incubation at 30 °C and to regularly shake the cells (30 s per hour). OD_620_ was recorded every 30 min for 24–30 h. Growth inhibition assays were performed in biological duplicates or triplicates. The half maximal inhibitory concentration (IC50) was determined as described previously using an end-point assay [65]. In brief, in order to generate a dose-response curve, the growth (OD_620_) of the treated cells normalised to the highest growth of the untreated control (OD max ctrl−) was plotted against the concentration in log_10_ units. Using the slope of the dose-response curve, the IC50 was determined as the point where the relative growth rate (OD 25 h/OD max ctrl−) was 0.5 (y = 0.5).

### Flow cytometry analysis for cell viability

Flow cytometry analysis of cell viability using BD Accuri C6 Plus flow cytometer equipped with a Csampler autosampler (Becton & Dickinson Biosciences, United States) was performed as described previously [66]. Cell samples were diluted to an OD620 between 0.1 and 0.4 in 0.5 ml of PBS buffer and stained with a SYBR Green/Propidium Iodide (PI) dye mix in order to differentiate between live and dead cells. Fluorescence excitation was made with a blue laser (λ = 488 nm), and the fluorescence emission of SYBR green and PI were collected at 533/30 nm (Fl1) and 585/40 nm (Fl3), respectively. The fluidics was set to fast flow rate, the threshold was set to 80,000 on the forward scatter channel, and 10,000 cells were collected. Data visualisation and analysis was made with the FlowJo™ Single Cell Analysis Software v10 (FlowJo, LLC, v10, Becton & Dickinson).

### Whole-cell bioconversion in bioreactors

Pre-cultures were prepared by inoculating a single colony from an agar plate into a baffled shake flask with 50 mL of defined mineral medium [59] with 20 g L^−1^ glucose, and buffered at pH 6.5. The pre-culture was incubated at 30 °C for at least 24 h at 180 rpm shake flask incubator (New Brunswick Scientific, New Jersey, USA). The preculture was used to inoculate a 1.4 L bench-top bioreactor (Multifors, Infors HT, Bottmingen, Switzerland) or a 3 L bench-top bioreactor (Minifors 2. Infors HT, Bottmingen, Switzerland) at OD = 0.2 in 500 mL of modified defined yeast mineral medium buffered at pH 6.5 (salt-, vitamin-, trace elements solutions), with 50 g L^−1^ glucose, supplemented with 0.5 g L^−1^ vanillylamine and 0.1 g L^−1^ nonanoic acid. The pH in the bioreactor was regulated at 6.5 by adding 3 M KOH, stirring was set at 300 rpm, the temperature was maintained at 30 °C and the bioreactor was aerated at 500 mL/min of air. Cell density was monitored by measuring optical density at 620 nm (OD_620_). Experiments were performed in biological duplicates or triplicates.

### Product extraction

Cells were separated from reaction broth by centrifugation at 5000 rpm for 10 min. The wet weight of the cell pellet was measured and the dialyzable yeast protein extraction reagent Y-PER™ Plus (Thermo Fisher Scientific Inc., Rockford, USA) with the addition of 0.1 M dithiotreitol (DTT; Thermo Scientific, USA), as instructed in the manual, was used to permeabilise the cells. Cell debris was separated by centrifugation at 5000 rpm for 10 min and the supernatant was collected. Supernatants were transferred to an extraction funnel and extracted with 1 or 2 volumes of ethyl acetate, for the reaction broth and cell extract respectively. The organic phase was transferred to a rotary glass flask and evaporated using a rotary vacuum evaporator. The resulting viscous oil was dissolved in 2 mL of methanol and further HPLC and LC-MS analysis were performed.

### Analytical methods

For the analysis of glucose and extracellular metabolites (ethanol, glycerol and acetate), a Waters HPLC system (Milford, USA) and a refractive index detector (Waters 2414, Milford, USA) were used. The column was an Aminex HPX-87 H ion-exchange column (7.8 × 300 mm, Bio-Rad, Hercules, USA) kept at 60 °C and the mobile phase was 5 mM H_2_SO_4_ with a flow rate of 0.6 mL/min (isocratic method).

For the analysis of nonivamide, a Waters HPLC system (Waters Binary HPLC pump 1525, UV/Vis detector 2489, Auto sampler 2707, All Waters Corporation, Milford, USA) with a Select C18 column (4.6 × 150 mm) was used. The analysis was run with reverse-phase chromatography and two mobile phases: millipore water with 0.1% trifluoroacetic acid (TFA) (solvent A) and acetonitrile (solvent B) in a gradient method for 30 min. The method started with an initial solvent composition of 65% of A and 35% of B and was kept for 5 min. Within the next 5 min, the composition was changed to 35% A and 65% B and that ratio was kept isocratic for 10 min. During the last 5 min of the method the ratio changed back to the initial conditions. The flow was set to 1 mL/min and the monitored wavelength at 281 nm. The analysis was performed at room temperature. A standard curve was made to calculate the concentration of the product in the sample.

Liquid chromatography–tandem mass spectrometry LC-MS/MS analysis was also performed to confirm the presence of nonivamide. The same column and mobile phase were used as already described above for the chromatographic separation. An Velos Pro Ion-trap MS system was used for identification by electrospray ionisation (ESI) in positive mode full scan as well as MS/MS experiments using CID fragmentation of the molecules [M+H]^+^ of the nonivamide standard (m/z = 294.204) and ion transitions (m/z 294 → 137) occurring in MS/MS, in which m/z 137 indicated the presence of a vanillyl moiety.

## Supplementary Information

**Additional file 1.** Gene sequences containing A. CoA-ligase sequences, B-Acyltransferase sequences. **Table S1.** Data from protein analysis using LC-MS/MS.

## Data Availability

The data generated and/or analyzed during this study is included in this article and Additional file. Additional information or material, such as plasmids and strains, are available upon request.

## Abbreviations

CoA: Coenzyme A
ATP: Adenosine triphosphate
CFU: Colony forming unit
ABC: ATP-binding cassette transporters
PCR: Polymerase chain reaction
HPLC: High-performance liquid chromatography
LC-MS/MS: Liquid chromatography–tandem mass spectrometry
