# Supplementary material for: Engineering Saccharomyces cerevisiae for production of the capsaicinoid nonivamide
Source: Microb Cell Fact. 2022 May 28;21:106. doi: 10.1186/s12934-022-01831-3 (PMC9148506; doi:10.1186/s12934-022-01831-3)
Supplement: Supplementary file 1 — Additional file 1. Gene sequences containing A. CoA-ligase sequences, B-Acyltransferase sequences. Table S1. Data from protein analysis using LC-MS/MS. [file 12934_2022_1831_MOESM1_ESM.pdf]

Appendix – Additional information

Gene sequences

A. CoA-ligase sequences

>Amino acid sequence ACS from *Capsicum annuum* (Uniprot B5LAV6)

MATDKFIIEVESAKPAKDGRPSMGPVYRSIFAKHGFPPPIPGLDSCWDIFRMSVEKYPNNRMLGRREI
VDGKPGKYVWMSYKEVYDIVIKVGNSIRSIGVDVGDKCGIYGANCPEWIISMEACNAHGLYCVPLYDT
LGAGAVEFIISHAEVTIAFVEEKKLPELLKTFPNASKYLKTIVSFGKVTPEQKKELEEFGVVLYSWDE
FLQLGSGKQFDLPVKKKEDICTIMYTSGTTGDPKGVLISNTSIVTLIAGVRRFLGSVDESLNVDDVYL
SYLPLAHIFDRVIEECFIHHGASIGFWRGDVKLLTEDIGELKPTVFCAVPRVLDRIYSGLQQKIAAGG
FLKSTLFNLAYAYKHHNLKKGRKHFEASPLSDKVVFSKVKEGLGGRVRLILSGAAPLAAHVEAFLRVV
ACCHVLQGYGLTETCAGTFVSLPNRYDMLGTVGPPVPNVDVCLESVPEMSYDALSSTPRGEVCVRGDI
LFSGYYKREDLTKEVMIDGWFHTGDVGEWQPNGSLKIIDRKKNIFKLSQGEYVAVENLENIYGNNPII
DSIWIYGNSFESFLVAVINPNQRAVEQWAEVNGLSGDFASLCEKPEVKEYILRELTKTGKEKKLKGFE
FLKAVHLDPVPFDMERDLLTPTFKKKRPQLLKYYKDVIDSMYKGTK

>Codon optimized nucleotide sequence ACS using ThermoScientific GeneArt®

ATGGCCACCGATAAGTTCATCATCGAAGTTGAATCTGCTAAGCCAGCTAAAGATGGTAGACCATCTAT
GGGTCCAGTTTACAGATCTATTTTCGCTAAACATGGTTTCCCACCACCAATTCCAGGTTTGGATTCTT
GTTGGGATATCTTCAGAATGTCCGTTGAAAAGTACCCAAACAACAGAATGTTGGGTAGAAGAGAAATC
GTTGATGGTAAACCAGGTAAGTACGTTTGGATGTCCTACAAAGAAGTTTACGACATCGTTATCAAGGT
CGGTAACTCCATTAGATCCATTGGTGTTGATGTTGGTGATAAGTGTGGTATCTATGGTGCTAATTGTC
CAGAATGGATCATTTCTATGGAAGCTTGTAATGCCCATGGTTTGTACTGTGTTCCATTATACGATACT
TTGGGTGCTGGTGCTGTTGAATTCATTATTTCTCATGCCGAAGTTACCATTGCCTTCGTTGAAGAAAA
AAAGTTGCCAGAATTATTGAAAACCTTCCCAAACGCCTCCAAGTACTTAAAGACTATCGTTTCTTTCG
GTAAGGTCACCCCAGAACAAAAGAAAGAATTGGAAGAATTCGGTGTCGTCTTGTACTCTTGGGATGAA
TTTTTACAATTGGGTTCCGGTAAGCAATTCGATTTGCCAGTTAAGAAGAAAGAAGATATCTGTACCAT
CATGTACACCTCTGGTACTACTGGTGATCCAAAAGGTGTTTTGATTTCCAACACTTCCATCGTTACTT
TGATTGCCGGTGTTAGAAGATTCTTGGGTTCTGTTGATGAATCCTTGAACGTTGATGATGTCTACTTG
TCTTATTTGCCATTGGCCCATATCTTCGATAGAGTCATTGAAGAATGCTTCATTCATCATGGTGCCTC
CATTGGTTTTTGGAGAGGTGATGTTAAGTTGTTGACCGAAGATATCGGTGAATTGAAGCCAACTGTTT
TTTGTGCTGTTCCAAGAGTTTTGGACAGAATCTACTCTGGTTTACAACAAAAGATTGCTGCTGGTGGT
TTCTTGAAGTCTACTTTGTTTAATTTGGCCTACGCCTACAAGCACCATAATTTGAAAAAGGGTAGAAA
GCACTTCGAAGCCTCTCCATTGTCTGATAAGGTTGTTTTCTCTAAGGTCAAAGAAGGTTTGGGTGGTA
GAGTTAGATTGATTTTGTCTGGTGCTGCTCCATTGGCTGCTCATGTTGAAGCTTTTTTGAGAGTTGTT

GCTTGTTGCCACGTTTTACAAGGTTATGGTTTGACTGAAACTTGCGCTGGTACTTTTGTTTCTTTGCC
AAACAGATACGATATGTTAGGTACTGTTGGTCCACCAGTTCCAAATGTTGATGTATGTTTGGAATCCG
TCCCAGAAATGTCTTATGATGCTTTATCTTCTACTCCAAGAGGTGAAGTTTGTGTTAGAGGTGATATT
TTGTTCTCCGGTTACTACAAGAGAGAAGATTTGACCAAAGAAGTCATGATTGACGGTTGGTTTCATAC
TGGTGACGTTGGTGAATGGCAACCTAATGGTTCTTTGAAGATCATCGACAGAAAAAAGAACATCTTCA
AGTTGTCCCAAGGTGAATACGTTGCTGTCGAAAATTTGGAAAACATCTACGGTAACAACCCAATCATC
GATTCCATTTGGATCTATGGTAACTCCTTCGAATCTTTCTTGGTTGCCGTTATCAACCCAAATCAAAG
AGCTGTTGAACAATGGGCTGAAGTTAATGGTTTGTCAGGTGATTTTGCTTCCTTGTGTGAAAAGCCAG
AAGTCAAAGAATATATCTTGAGAGAATTGACCAAGACCGGTAAAGAAAAGAAATTGAAGGGTTTCGAA
TTCTTGAAGGCCGTTCACTTGGATCCAGTTCCATTTGATATGGAAAGAGATTTGTTGACCCCAACTTT
CAAGAAGAAAAGACCACAATTATTGAAGTACTACAAGGACGTCATCGACTCTATGTACAAAGGTACTA
AGTAA

>Amino acid sequence PhCL from *Petunia hybrid* (Uniprot I3PB37)

MPMETETNQGDLIFRSKLPDIYIPKHLPLHSYCFENISEFSSRPCLINGANNHIYTYADVELTSRKVA
AGLNKLGIQQKDTIMILLPNSPEFVFAFMGASYLGAISTMANPLFTPAEVVKQAKASNAKLIITQACF
VNKVKDYAFDNNLNVICIDSAPEGCIHFSELTQADEHDIPDVKIQSDDVVALPYSSGTTGLPKGVMLT
HKGLVTSVAQQVDGENANLYMHSEDVLMCVLPLFHIYSLNSVLLCGLRVGAAILIMQKFDIVQFCELI
EKYKVTIGPFVPPIVLAIAKSPVVDNYDLSSVRTVMSGAAPLGKELEDAVRIKFPNAKLGQGYGMTEA
GPVLAMCLAFAKEPFDIKSGACGTVVRNAEMKIVDPDTGCSLPRNQPGEICIRGDQIMKGYLNDPAAT
TRTIDKEGWLHTGDIGYIDNDDELFIVDRLKELIKYKGFQVAPAELEALLLNHPNISDAAVVPMKDEQ
AGEVPVAFVVRSNGSDITEDEVKDFVSKQVIFYKRIKRVFFVETVPKSPSGKILRKDLRARLAAGVPN

>Codon optimized nucleotide sequence PhCL using ThermoScientific GeneArt®

ATGCCAATGGAAACCGAAACTAACCAGGGTGATTTGATCTTCAGATCTAAGTTGCCAGATATCTACAT
CCCAAAACATTTGCCATTGCACTCTTACTGCTTCGAAAACATCTCTGAATTCTCTTCTAGACCCTGCT
TGATTAACGGTGCTAACAATCATATCTACACCTACGCTGATGTTGAATTGACCTCTAGAAAAGTTGCT
GCTGGTTTGAACAAGTTGGGTATTCAACAAAAGGACACCATCATGATCTTGTTGCCAAATTCTCCAGA
ATTCGTGTTCGCTTTTATGGGTGCTTCTTATTTGGGTGCTATTTCTACTATGGCTAACCCTTTGTTTA
CTCCAGCTGAAGTTGTTAAGCAAGCTAAGGCTTCTAACGCCAAGTTGATTATTACTCAAGCTTGCTTC
GTTAACAAGGTTAAGGATTACGCTTTCGACAACAACTTGAACGTTATCTGCATTGATTCTGCTCCAGA
AGGTTGCATTCACTTCTCAGAATTGACTCAAGCTGATGAACATGATATCCCAGATGTCAAGATCCAAT
CCGATGATGTTGTTGCTTTGCCATATTCTTCAGGTACTACTGGTTTGCCAAAAGGTGTTATGTTGACT
CACAAAGGTTTGGTTACATCCGTTGCTCAACAAGTTGATGGTGAAAATGCTAACTTGTACATGCACTC
CGAAGATGTTTTGATGTGTGTTTTGCCTCTGTTCCACATCTACTCATTGAACTCTGTTTTGTTGTGCG
GTTTGAGAGTTGGTGCTGCTATTTTGATTATGCAGAAGTTCGATATCGTCCAGTTCTGCGAATTGATC
GAAAAGTACAAGGTTACCATTGGTCCATTTGTTCCACCAATCGTTTTGGCTATTGCTAAGTCTCCAGT

TGTTGACAACTACGATTTGTCCTCTGTTAGAACTGTTATGTCAGGTGCTGCTCCATTGGGTAAAGAAT
TGGAAGATGCTGTTAGAATCAAGTTCCCAAACGCTAAATTAGGTCAAGGTTACGGTATGACTGAAGCT
GGTCCAGTTTTAGCTATGTGTTTGGCATTTGCTAAAGAGCCATTCGATATTAAGTCTGGTGCTTGTGG
TACTGTTGTTAGAAATGCTGAAATGAAGATCGTTGATCCAGATACCGGTTGTTCATTGCCAAGAAATC
AACCAGGTGAAATCTGCATTAGAGGTGACCAAATTATGAAGGGTTACTTGAATGATCCAGCTGCTACT
ACTAGAACCATTGACAAAGAAGGTTGGTTGCATACTGGTGATATCGGTTACATTGATAACGATGACGA
GTTGTTCATCGTCGACAGATTGAAAGAGTTGATCAAGTATAAGGGTTTCCAAGTTGCTCCTGCTGAAT
TAGAAGCTTTGTTGTTGAACCATCCAAACATTTCTGATGCTGCTGTTGTTCCAATGAAGGATGAACAA
GCTGGTGAAGTTCCAGTTGCTTTCGTTGTTAGATCAAACGGTTCTGATATCACCGAAGATGAAGTTAA
GGACTTCGTTTCCAAGCAAGTCATTTTCTACAAGAGGATCAAGAGGGTTTTCTTCGTTGAAACTGTTC
CAAAATCTCCATCCGGTAAGATCTTGAGAAAAGATTTGAGAGCTAGATTGGCAGCTGGTGTTCCAAAC
TAAATTGAATTGAATTGAAATCGATAGATCAATTTTTTTCTTTTCTCTTTCCCGAGCTC

<u>>Amino acid sequence ipfF from *Sphingomonas Ibu-2* (Uniprot A1E027)</u>

MLARDLVKRCARNYPTKTAYLCGERSRSWREMDQRSDRFGVALQQLGHRPGEAVAILTQESIEVYEHF
FACMKIAAPRVGLNTGYVWPEMLHVLKDSEVKFLLLDTRCRHLLAERLGELKALGITLIGYGAGHGLE
RDYESLLATAEGEPHWPALAPDDILFVSYTSGTTGVPKGVMLTQEGGVNCILHSLISFGFGPDDVWYM
PAASAWVVVILNAFGLGNGMTTVIPDGGYQLQAYLRDIERFRVTVGMLVPTMLQRAIVEIQTNPVYDL
SSLRMVVYGSSPATPKLIRDARATFKGIKLLQAYAMTEATGGWISYLTDADHEHALREEIELLKSVGR
IGIHYDCSIRDESGQPVPIGQSGEIWLRGNTMMKGYRNLPEATAEAMPDGWLRTNDIGRLDERGYLYL
LDRQKFLIITGAVNVFPTTVEAILVEHPAVEEVAVVGVPHPEWGEAVVAVVVRKPSHRDVTVQALIDF
CHGKLSRPETPKHVVFVDELPKTSNAKLKKGELKKWLSGGAVPLPWQLEVA

<u>>Codon optimized nucleotide sequence ipfF using ThermoScientific GeneArt®</u>

ATGTTGGCCAGAGATTTGGTTAAGAGATGCGCTAGAAATTACCCAACTAAGACTGCTTATTTGTGCGG
TGAAAGATCTAGAAGTTGGAGAGAAATGGATCAGAGATCTGATAGATTTGGTGTTGCCTTGCAACAAT
TGGGTCATAGACCAGGTGAAGCTGTTGCTATTTTGACCCAAGAATCCATCGAAGTTTACGAACATTTC
TTCGCCTGTATGAAGATTGCTGCTCCAAGAGTTGGTTTGAATACTGGTTATGTTTGGCCAGAAATGTT
GCACGTTTTGAAGGATTCTGAAGTCAAGTTCTTGTTGTTGGATACCAGATGCAGACATTTGTTGGCTG
AAAGATTGGGTGAATTGAAGGCTTTGGGTATTACCTTGATTGGTTATGGTGCTGGTCATGGTTTGGAA
AGAGATTACGAATCTTTGTTGGCAACTGCTGAAGGTGAACCACATTGGCCAGCTTTGGCTCCAGATGA
TATTTTGTTTGTTTCTTACACCTCTGGTACTACCGGTGTTCCAAAAGGTGTTATGTTGACTCAAGAAG
GTGGCGTTAACTGTATCTTGCACTCTTTGATTTCTTTCGGTTTTGGTCCTGATGATGTCTGGTATATG
CCAGCTGCTTCTGCTTGGGTTGTTGTTATATTGAACGCTTTTGGTCTAGGTAACGGTATGACTACTGT
TATTCCAGATGGTGGTTATCAATTGCAAGCCTACTTGAGAGATATCGAAAGATTCAGAGTTACCGTTG
GTATGTTGGTTCCAACTATGTTGCAAAGAGCCATCGTTGAAATTCAAACTAACCCAGTTTACGACCTG
TCCTCTTTGAGAATGGTTGTTTATGGTTCTTCTCCAGCTACTCCAAAGTTGATTAGAGATGCTAGAGC

TACTTTCAAGGGCATCAAATTATTGCAAGCTTACGCTATGACTGAAGCTACTGGTGGTTGGATTTCTT
ATTTGACTGATGCCGATCATGAACACGCTTTGAGAGAAGAAATTGAGTTGTTGAAGTCCGTTGGTAGA
ATCGGTATTCATTACGATTGCTCCATCAGAGATGAATCTGGTCAACCAGTTCCAATTGGTCAATCTGG
TGAAATTTGGTTGAGAGGTAACACTATGATGAAGGGCTATAGAAATTTGCCAGAAGCTACAGCTGAAG
CTATGCCTGATGGTTGGCTAAGAACTAATGATATTGGTAGATTGGACGAGAGGGGTTACTTGTACTTG
TTAGATAGACAGAAGTTCTTGATTATCACCGGTGCTGTTAATGTTTTCCCAACTACTGTTGAAGCCAT
CTTGGTTGAACATCCAGCTGTTGAAGAGGTTGCTGTTGTTGGTGTTCCACATCCAGAATGGGGTGAAG
CAGTTGTTGCTGTAGTTGTTAGAAAACCATCTCACAGAGATGTTACCGTTCAAGCCTTGATTGATTTC
TGCCATGGTAAATTGTCTAGGCCAGAAACTCCAAAGCACGTTGTTTTTGTTGATGAATTGCCAAAGAC
CTCCAACGCTAAATTGAAGAAAGGTGAGTTGAAAAAGTGGTTGTCTGGTGGTGCTGTTCCATTGCCTT
GGCAATTGGAAGTTGCTTAA

B. Acyltransferase sequences

<u>>Amino acid sequence AT3 from *Capsicum chinense* (Uniprot Q58VT0)</u>

MAFALPSSLVSVCDKSFIKPSSLTPSKLRFHKLSFIDQSLSNMYIPCAFFYPKVQQRLEDSKNSDELS
HIAHLLQTSLSQTLVSYYPYAGKLKDNATVDCNDMGAEFLSVRIKCSMSEILDHPHASLAESIVLPKD
LPWANNCEGGNLLVVQVSKFDCGGIAISVCFSHKIGDGCSLLNFLNDWSSVTRDHTTTALVPSPRFVG
DSVFSTKKYGSLITPQILSDLNECVQKRLIFPTDKLDALRAKVAEESGVKNPTRAEVVSALLFKCATK
ASSSMLPSKLVHFLNIRTMIKPRLPRNAIGNLSSIFSIEATNMQDMELPTLVRNLRKEVEVAYKKDQV
EQNELILEVVESMREGKLPFENMDGYENVYTCSNLCKYPYYTVDFGWGRPERVCLGNGPSKNAFFLKD
YKAGQGVEARVMLHKQQMSEFERNEELLEFIA

<u>>Codon optimized nucleotide sequence AT3 using ThermoScientific GeneArt®</u>

ATGGCTTTCGCTTTGCCATCCTCTTTGGTTTCTGTTTGTGATAAGTCCTTCATCAAGCCATCTTCATT
GACTCCATCTAAGTTGAGATTCCACAAGTTGTCTTTCATCGATCAATCCTTGTCCAACATGTACATTC
CATGTGCATTTTTCTACCCAAAGGTTCAACAAAGATTGGAAGATTCCAAGAACTCCGATGAATTGTCC
CATATTGCTCACTTGTTGCAAACCTCATTGTCTCAAACATTGGTCAGTTATTATCCATACGCCGGTAA
GTTGAAGGATAATGCTACTGTTGATTGCAACGATATGGGTGCTGAATTCTTGTCTGTTAGAATCAAGT
GCTCCATGTCCGAAATTTTGGATCATCCACATGCTTCATTGGCCGAATCTATAGTTTTGCCAAAAGAT
TTGCCATGGGCTAACAATTGTGAAGGTGGTAATTTGTTGGTTGTCCAAGTTTCTAAGTTCGATTGTGG
TGGTATTGCTATCTCTGTTTGCTTCTCTCATAAGATTGGTGATGGTTGCTCCTTATTGAACTTCTTGA
ATGATTGGTCCTCCGTTACTAGAGATCATACAACTACTGCTTTGGTTCCATCTCCAAGATTCGTTGGT
GATTCTGTTTTCTCTACTAAGAAGTACGGTTCCTTGATTACCCCACAAATCTTGTCTGATTTGAACGA
ATGTGTCCAAAAGAGATTGATTTTCCCAACCGATAAGTTGGATGCTTTGAGAGCTAAAGTTGCTGAAG
AATCTGGTGTTAAGAATCCAACTAGAGCCGAAGTTGTTTCTGCTTTGTTGTTTAAGTGTGCTACCAAG
GCTTCTTCTTCTATGTTGCCATCTAAATTGGTCCATTTCTTGAACATCAGAACCATGATCAAACCTAG

ATTGCCAAGAAACGCCATTGGTAACTTGTCCTCCATTTTCTCTATTGAAGCTACCAACATGCAAGATA
TGGAATTGCCAACTTTGGTCAGAAACTTGAGAAAAGAAGTTGAAGTCGCCTACAAGAAGGATCAAGTT
GAACAAAACGAATTGATCTTGGAAGTCGTTGAATCCATGAGAGAAGGTAAATTGCCATTCGAAAACAT
GGACGGTTACGAAAACGTTTACACCTGTTCTAATTTGTGCAAGTACCCTTACTACACCGTTGATTTTG
GTTGGGGTAGACCAGAAAGAGTTTGTTTGGGTAACGGTCCATCTAAAAACGCATTCTTTTTGAAGGAT
TACAAGGCCGGTCAAGGTGTTGAAGCTAGAGTTATGTTGCATAAGCAACAAATGTCCGAATTCGAAAG
AAACGAAGAATTATTGGAATTCATTGCTTAA

>Amino acid sequence PaAT from *Pseudomonas aeruginosa* (Uniprot Q9HUY3)

MTPLTPEQTHAYLHHIGIDDPGPPSLANLDRLIDAHLRRVAFENLDVLLDRPIEIDADKVFAKVVEGS
RGGYCFELNSLFARLLLALGYELELLVARVRWGLPDDAPLTQQSHLMLRLYLAEGEFLVDVGFGSANP
PRALPLPGDEADAGQVHCVRLVDPHAGLYESAVRGRSGWLPLYRFDLRPQLWIDYIPRNWYTSTHPHS
VFRQGLKAAITEGDLRLTLADGLFGQRAGNGETLQRQLRDVEELLDILQTRFRLRLDPASEVPALARR
LAGLISA

>Codon optimized nucleotide sequence PaAT using ThermoScientific GeneArt®

ATGACCCCATTGACTCCAGAACAAACTCATGCTTACTTGCATCATATCGGTATTGATGATCCAGGTCC
ACCATCTTTGGCTAATTTGGATAGATTGATTGATGCCCACTTGAGAAGAGTTGCTTTCGAAAATTTGG
ATGTCTTGTTGGACAGACCAATTGAAATTGATGCCGATAAGGTTTTCGCCAAGGTTGTTGAAGGTTCT
AGAGGTGGTTACTGTTTCGAATTGAATTCTTTGTTCGCCCGTTTGTTGTTGGCTTTGGGTTATGAATT
GGAACTGTTGGTTGCTAGAGTTAGATGGGGTTTGCCAGATGATGCTCCATTAACTCAACAATCCCATT
TGATGTTGAGGTTGTATTTGGCTGAAGGTGAATTCTTGGTTGATGTTGGTTTTGGTTCTGCTAATCCA
CCAAGAGCTTTGCCATTGCCAGGTGATGAAGCTGATGCTGGTCAAGTTCATTGTGTTAGATTGGTTGA
TCCACATGCTGGCTTGTATGAATCTGCTGTTAGAGGTAGATCTGGTTGGTTGCCATTATACAGATTTG
ATTTGCGTCCACAGTTGTGGATTGATTACATTCCAAGAAACTGGTACACTTCTACCCATCCACATTCT
GTTTTTAGACAAGGTTTGAAGGCTGCTATTACCGAAGGTGATTTGAGATTGACTTTAGCCGATGGTTT
GTTTGGTCAACGTGCTGGTAATGGTGAAACATTGCAAAGACAATTGAGAGATGTCGAAGAGTTGTTGG
ATATCTTGCAAACCAGATTCAGATTGAGATTGGATCCAGCTTCTGAAGTTCCAGCTTTGGCTAGAAGA
TTGGCTGGTTTGATTTCTGCTTGA

>Amino acid sequence CaAT from *Capsicum annuum* (Uniprot Q5D8C0)

MASAISETITTNGPSENNNLTITGKIHTRVRLATKSDLHHIYQLFYQIHAYHNFTHLYKATESSLGDL
LFKENPLPLFYGPSVLLLEVSPTPFTQPKNNKDEGFKPVLTTFNLKFPVVEGQVEEFQSKYDDGNDKR
DVFIAGYAFFYANYSCFYDKPGFYFESLYFRESYRKLGMGRLLFGTVASIAANNGFVSVEGIVAVWNK
KSYDFYIDMGVEIFDEFRYGKLHGENLQKYADKQKNEGGNC

<u>>Codon optimized nucleotide sequence CaAT using ThermoScientific GeneArt®</u>

ATGGCCTCCGCTATTTCTGAAACTATTACTACTAATGGCCCCTCCGAAAACAACAACTTGACTATTAC
TGGTAAGATCCACACCAGAGTTAGATTGGCTACAAAATCTGACTTGCATCACATCTACCAGTTGTTCT
ACCAAATTCATGCCTACCATAACTTCACCCACTTGTACAAAGCTACCGAATCTTCTTTGGGTGACTTG
TTGTTTAAAGAGAACCCATTGCCTTTGTTCTACGGTCCATCTGTTTTGTTGTTGGAAGTTTCTCCAAC
TCCATTCACTCAACCTAAGAACAACAAGGATGAAGGTTTCAAGCCAGTTTTGACTACCTTCAATTTGA
AGTTCCCAGTTGTTGAAGGTCAGGTCGAAGAATTTCAATCCAAATACGATGATGGTAACGATAAGAGG
GATGTTTTCATTGCTGGTTACGCTTTTTTCTACGCTAACTACTCTTGCTTCTATGACAAGCCAGGTTT
CTACTTCGAATCCTTGTACTTCAGAGAGTCCTACAGAAAATTAGGTATGGGTAGATTGCTGTTCGGTA
CTGTTGCTTCAATTGCTGCTAACAATGGTTTCGTTTCAGTTGAAGGTATAGTTGCCGTTTGGAACAAA
AAGTCCTACGATTTCTACATCGATATGGGTGTCGAAATCTTCGACGAATTCAGATACGGTAAATTGCA
CGGTGAAAACTTGCAAAAGTACGCCGATAAGCAAAAGAATGAAGGTGGTAACTGTTAA

<u>>Amino acid sequence SlAT from *Solanum lycopersicum* (Uniprot Q8RXB8)</u>

MAPALEQAITSDASSDVTITGKIYTRVRLATKSDLSHIYRLFYQIHEYHNYTHLYKATESSLANLLFK
ENPLPLFYGPSVLLLEVSPTPFDEPKNTTDEGFKPVLTTFDLKFPVVEGEVEEFRSKYDDKSDVYIAG
YAFFYANYSCFYDKPGFYFESLYFRESYRKLGMGSLLFGTVASIAANNGFVSVEGIVAVWNKKSYDFY
VNMGVEIFDEFRYGKLHGENLQKYAHN

<u>>Codon optimized nucleotide sequence SlAT using ThermoScientific GeneArt®</u>

ATGGCTCCAGCTTTGGAACAAGCTATTACTTCTGATGCTTCTTCCGATGTTACCATCACTGGTAAAAT
CTACACCAGAGTTAGATTGGCTACCAAGTCTGATTTGTCTCATATCTACAGGTTGTTCTACCAGATCC
ATGAATACCATAACTACACCCACTTGTACAAAGCTACCGAATCTTCTTTGGCTAACCTGTTGTTTAAA
GAGAACCCATTGCCTTTGTTTTACGGTCCATCTGTTTTGTTGTTGGAAGTTTCTCCAACTCCATTCGA
TGAACCTAAGAACACTACTGATGAAGGTTTCAAGCCAGTTTTGACTACCTTCGATTTGAAGTTCCCAG
TTGTTGAAGGTGAAGTCGAAGAATTCAGATCTAAGTACGATGATAAGTCCGATGTTTACATTGCTGGT
TACGCTTTTTTCTACGCTAACTACTCTTGCTTCTATGACAAGCCAGGTTTCTACTTCGAATCCTTGTA
CTTCAGAGAGTCCTACAGAAAATTAGGTATGGGCTCTTTGTTGTTCGGTACTGTTGCTTCAATTGCTG
CTAACAATGGTTTCGTTTCAGTTGAAGGTATAGTTGCTGTCTGGAACAAAAAGTCCTACGATTTCTAC
GTTAACATGGGTGTTGAAATCTTCGACGAGTTCAGATACGGTAAATTGCATGGTGAAAACTTGCAAAA
GTACGCCCATAACTGA

Data from protein analysis using LC-MS/MS

Table S1. Relevant information regarding the ACS and AT3 proteins detected from the proteomics experiment

			Strain			
	Protein		TMBNM009		TMBNM020	
	ACS	AT3	ACS	AT3	ACS	AT3
Peptides	32.0	11.0	32.0	10.0	26.0	6.0
Sequence coverage [%]	52.6	30.2	52.6	28.4	32.7	14.3
MS/MS count	141.0	16.0	70.0	10.0	71.0	6.0
Intensity	5.0E+10	1.1E+09	3.7E+10	8.3E+08	1.3E+10	2.5E+08
LFQ intensity			4.2E+09	2.5E+08	3.0E+09	1.5E+08
Mol. weight [kDa]	73.6	49.3				
Sequence length	658.0	440.0				
Q-value	0.0	0.0				
Score	323.3	100.7				
